# Nerve ablation after bronchial thermoplasty and sustained improvement in severe asthma

**DOI:** 10.1186/s12890-017-0554-8

**Published:** 2018-02-08

**Authors:** N. Facciolongo, A. Di Stefano, V. Pietrini, C. Galeone, F. Bellanova, F. Menzella, N. Scichilone, R. Piro, G. L. Bajocchi, B. Balbi, L. Agostini, P. P. Salsi, D. Formisano, M. Lusuardi

**Affiliations:** 1 Department of Medical Specialties, Pulmonology Unit, Arcispedale Santa Maria Nuova – IRCCS, Azienda USL di Reggio Emilia, Reggio Emilia, Italy; 2 Pulmunology Unit and Laboratory of Citoimmunopatology, Istituti Clinici Scientifici Maugeri SpA, SB, IRCCS, Veruno (NO), Italy; 3 Department of Neurosciences, Laboratory of Neuropathology, University of Parma, Parma, Italy; 4 Rheumatology Unit, Department of Internal Medicine, Azienda Ospedaliera ASMN, Istituto di Ricovero e Cura a Carattere Scientifico, Reggio Emilia, Italy; 5 Anesthesiology and Critical Care Unit, Arcispedale Santa Maria Nuova -IRCCS, Azienda USL di Reggio Emilia, Reggio Emilia, Italy; 6 Research and Statistics, Arcispedale Santa Maria Nuova -IRCCS, Azienda USL di Reggio Emilia, Reggio Emilia, Italy; 7 Pulmonary Rehabilitation, S. Sebastiano Hospital, Correggio (RE), Azienda USL di Reggio Emilia, Reggio Emilia, Italy; 8 Departement of Biomedicine and Medical Specialties, Pulmonology Unit, University of Palermo, Palermo, Italy

**Keywords:** Bronchial thermoplasty, Bronchial biopsies, Severe asthma, Nerve fibers, Bronchoscopy

## Abstract

**Background:**

Bronchial thermoplasty (BT) is a non-pharmacological intervention for severe asthma whose mechanism of action is not completely explained by a reduction of airway smooth muscle (ASM). In this study we analyzed the effect of BT on nerve fibers and inflammatory components in the bronchial mucosa at 1 year.

**Methods:**

Endobronchial biopsies were obtained from 12 subjects (mean age 47 ± 11.3 years, 50% male) with severe asthma. Biopsies were performed at baseline (T0) and after 1 (T1), 2 (T2) and 12 (T12) months post-BT, and studied with immunocytochemistry and microscopy methods. Clinical data including Asthma Quality of Life Questionnaire (AQLQ) and Asthma Control Questionnaire (ACQ) scores, exacerbations, hospitalizations, oral corticosteroids use were also collected at the same time points.

**Results:**

A statistically significant reduction at T1, T2 and T12 of nerve fibers was observed in the submucosa and in ASM compared to T0. Among inflammatory cells, only CD68 showed significant changes at all time points. Improvement of all clinical outcomes was documented and persisted at the end of follow up.

**Conclusions:**

A reduction of nerve fibers in epithelium and in ASM occurs earlier and persists at one year after BT. We propose that nerve ablation may contribute to mediate the beneficial effects of BT in severe asthma.

**Trial registration:**

Registered on April 2, 2013 at ClinicalTrials.gov Identifier: NCT01839591.

## Background

In the severe forms of asthma, airway smooth muscle (ASM) is believed to play an important role as a target for the neurogenic stimuli and inflammatory cytokines [1, 2]. Bronchial thermoplasty (BT) is a novel intervention for asthma whereby controlled thermal energy (65 °C) is delivered to the airway wall, resulting in a persistent reduction in airway smooth muscle (ASM) mass [3, 4]. Decreasing the amount and/or contractility of ASM may be a way to relieve symptoms and improve the quality of life of asthmatics [5–7]. The mechanism of action of BT is still largely unknown; indeed, the denaturation and destruction of the smooth muscle layer alone does not fully explain its effects [8, 9], and alternative mechanisms have been hypothesized. Recently, Pretolani et al. demonstrated that BT down-regulates selectively structural abnormalities involved in airway narrowing and bronchial reactivity, such as ASM, neuroendocrine epithelial cells and bronchial nerve endings as early as at 3 months after BT [10, 11], suggesting the occurrence of multiple mechanisms through which BT may exert its clinical efficacy. In this respect, the activation of local sensory nerve C-fibers by endogenous Transient Receptor Potential Vanilloid type 1 (TRPV1) activators or inhaled irritants may play a role in the complex mechanism of airway inflammation through the release of tachykinins [12], thus causing ASM contractility. The aim of our study was to explore the effect of BT on the nerve fiber network and whether it persisted after 1-year treatment.

## Methods

Patients with severe uncontrolled asthma as defined by GINA and ATS/ERS [1, 2] guidelines who underwent BT between March 2013 and July 2014 were enrolled at the Pneumology Unit of Azienda Ospedaliera ASMN-IRCCS Reggio Emilia, Italy. Subjects had to be free from exacerbation in the previous 3 weeks, defined as a worsening of asthma symptoms requiring a course or an increase of oral corticosteroids (OCS). Selection criteria are reported in Table 1. The study conformed to the Declaration of Helsinki and was approved by the institutional ethics committee (protocol n°2331, Reggio Emilia, February 18, 2013) and registered on *April 2,2013* at *ClinicalTrials.gov* *Identifier NCT0183959*. Written informed consent was obtained from each participant prior to enrolment in the study. All subjects underwent lung function evaluation according to ATS/ERS recommendations [13] at baseline, at 3 and 12 months post-BT. Type and timing of functional and clinical evaluations are reported in Table 2. Days of work lost registered in the twelve months before enrolment were compared with days of work lost in the year after BT.

**Table 1:** Selection criteria of the study population

Inclusion Criteria
1. Patients with severe persistent asthma, stable for at least 3 weeks
2. Patients receiving regular treatment with inhaled corticosteroids (beclomethasone ≥1000 mcg or equivalent) and long-acting ß agonists (LABA) (salmeterol ≥100 mcg or equivalent)
3. Asthma Quality of Life Questionnaire (AQLQ) score < 6.25
4. Asthma Control Questionnaire (ACQ) score > 2.0
5. Post-bronchodilator Forced Expiratory Volume in 1 s (FEV_1_) ≥ 60% predicted 6. Patients not smoking for at least one year
Exclusion Criteria
1. Life threatening asthma exacerbation
2. Concomitant respiratory diseases (e.g. COPD or emphysema)
3. Use of ß-blocker drugs
4. Severe active infection in the last 2 weeks
5. Pacemaker, internal defibrillator or other implanted electronic device
6. Known sensitivity to medications used to perform bronchoscopy, including lidocaine, atropine and benzodiazepines
7. Current uncontrolled bleeding disorders
8. Inability to stop taking, prior to the procedure, anticoagulants, antiplatelet agents, aspirin or non-steroidal anti-inflammatory drugs
9. Age < 18 years
10. Pregnancy

**Table 2:** Timing of functional and clinical evaluations and endoscopic procedures

	enrolment	BT 1° Baseline	BT 2° 1 month after T0	BT3° 2 months after T0	Follow-up 3 months after T2	Follow-up 12 months after T2
		T0	T1	T2	T3	T12
Consent	X					
Spirometry	X				X	X
ACQ	X				X	X
AQLQ	X				X	X
Drug Treatment		X	X	X		X
Safety		X	X	X	X	X
Exacerbations		X				X
Hospitalizations		X				X
BT		Treatment of right lower lobe	Treatment of left lower lobe	Treatment of right upper lobe Treatment of left upper lobe		
Biopsies		Biopsy of left lower lobe	Biopsy of right lower lobe	Biopsy of right lower lobe		Biopsy of right lower lobe

Legend: *T0* baseline, *T1* one month after T0; *T2* two months after T0, *T3* three months after T2, *T12* twelve months after T2 (i.e. 14 months after baseline)

### Fiberoptic bronchoscopy, BT and bronchial biopsies

Endoscopic procedures were carried out on spontaneous breathing under deep sedation with remifentanil 0.10 mcg/Kg/h and propofol 8 mg/Kg/h administered under anesthesiology assistance. Oxygen was administered via ventimask, FiO_2_ 50%. Oxygen saturation, blood pressure and electrocardiogram (ECG) were monitored with a Dynascope DS-7100 (Fukuda Denshi, Tokyo, Japan). BT was performed with a fiberoptic bronchoscope (Olympus BF-160, Tokyo, Japan) following standard procedures with an average of 75 ± 20 valid applications per lobe using the Alair catheter (Bronchial Thermoplasty System Model ATS2–5 and Model ATS200 Boston Scientific Corporation, Marlborough, MA, USA) [5–7]. The sequence of endoscopic procedures is summarized in Table 2. In detail, BT consisted of three separate sessions, each at one month interval from the other. The right and left lower lobes were treated in the first and second BT sessions, respectively; the two upper lobes were treated in the third session. As by convention, the middle lobe was not treated for the theoretical risk of stenosis because of its small diameter. At the end of each procedure and 12 months after BT (T12), 6 to 8 biopsies on the segmental and subsegmental bronchial carina were obtained. Care was taken to avoid sampling in areas showing visual effects of previous biopsies.

### Immunohistochemistry for nerve fiber evaluation

Three to four biopsies per patient per time point (T0, T1, T2, T12) were considered for nerve fiber immunohistochemistry and quantitation. Specimens were immediately fixed in cold periodate-lysine-paraformaldehyde (PLP) for up to 24 h at 4 °C, then kept in a cryoprotectant solution containing glycerol for one night and serially cut with a cryostat. Six serial sections (50 μm) were cut from each biopsy perpendicularly to the bronchial surface, therefore 6 sections × 3–4 biopsies (for a total of 18–24 sections per time point) were evaluated.

Free-floating sections were stained using primary antibodies raised in rabbit against the pan-axonal marker protein gene product 9.5 (PGP9.5; Bio-Rad Laboratories, Hercules, CA, USA; dilution 1:1200) species-specific biotinylated secondary antibody (Vector Lab Inc., Burlingame, CA, USA), Peroxidase Avidin Biotin Complex (ABC, Vector Labs, Burlingame, CA, USA), and peroxidase substrate Vector SG (Vector Labs). On the basis of morphology and overall area available, 2–3 best sections were selected from each biopsy and each time point. These sections were scored (0 to 3 values) for nerve fibers and the final score reported was the highest one for each time point determined in the semi-quantitative analysis carried out by two independent expert observers, in a blinded fashion, using bright field light microscopy at 40× magnification.

The number of nerve fibers was assessed in four different areas: 1) bronchial epithelium, 2) submucosa, 3) smooth muscle and 4) glands. Nerve fibers were arbitrarily scored as follows: 0 = absence of nerve fibers; 1 = occasional presence of nerve fibers, cut-off ≤3/mm^2^; 2 = few nerve fibers not uniformly distributed, cut-off >3 ≤ 15/mm^2^; 3 = numerous nerve fibers, uniformly distributed, cut-off >15/mm^2^.

### Immunohistochemistry for inflammatory cells

At least two samples were embedded in Tissue Tek II OCT (Miles Scientific, Naperville, IL, USA), frozen within 15 min in isopentane pre-cooled in liquid nitrogen, and stored at −80 °C. Six μm thick cryostat sections were cut for immunohistochemical light microscopy analysis and processed as described below.

One cryostat section from the best selected and oriented biopsy was stained for each inflammatory molecule studied applying immunohistochemical methods with a panel of mouse monoclonal antibodies specific for inflammatory and endothelial cells: CD45+/M701/dilution 1:100, CD4+/M716/1:200, CD8+/M7103/1:200, CD68+/M876/1:100, neutrophil elastase+/M752/1:100, mast cells (tryptase+)/M7052/1:300, endothelial cells (CD31+)/M823/1:40, from Dako Italia (Milan, Italy) and eosinophils (EG2+)/EG2/1:100 from Kabi Pharmacia (Uppsala, Sweden).

Briefly, for inflammatory cells, after blocking non-specific binding sites with serum derived from the same animal species as the secondary antibody, primary antibody was applied at optimal dilutions in TRIS-buffered saline (0.15 M saline containing 0.05 M TRIS-hydrochloric acid at pH 7.6) and incubated for 1 h at room temperature in a humid chamber. Antibody binding was demonstrated with secondary anti-mouse (Vector, BA 2000) antibodies followed by ABC kit AP AK5000, Vectastain and fast-red substrate (red color) or ABC kit HRP Elite, PK6100, Vectastain and diaminobenzidine substrate (brown color). The revealing system included a biotinylated secondary antibody (Vector Labs), ABC Complex (Vector Labs) and a peroxidase substrate Vector SG (Vector Labs). Nasal polyps were used as positive controls. For the negative control, normal mouse or rabbit non-specific immunoglobulins (Santa Cruz Biotechnology, Santa Cruz, CA, USA) were used at the same protein concentration as the primary antibody.

Morphometric measurements were performed with a light microscope (Leitz Biomed, Leica, Cambridge, UK) connected to a video recorder linked to a computerized image system (Quantimet 500 Image Processing and Analysis System, Software Qwin V0200B, Leica). Light-microscopic analysis was performed at a magnification of 630×.

Immunostained cells in the submucosa were quantified 100 μm beneath the epithelial basement membrane in subsequent non-overlapping high-power fields until the whole specimen was examined. The final result, expressed as the number of positive cells per square millimeter, was calculated as the average of all the cellular counts performed in each biopsy.

### Statistical analysis

Descriptive statistics were performed to investigate the sample characteristics; mean ± standard deviation, or median and interquartile interval (IQR) were chosen to summarize continuous variables, while absolute and relative frequencies (n, %) were used for categorical variables. Differences among time points were analyzed using repeated measures analysis of variance (ANOVA) for functional data or Friedman test (corrected for missing values) for immunohistochemical data. When repeated measures ANOVA was statistically significant, the post hoc tests with Bonferroni correction were performed. The Friedman test applied for morphologic data multiple comparison was followed by the Wilcoxon rank test for comparison between time points (T0, T1, T2, T12). Paired data Student’s t-test was applied to compare days of work lost before and after BT. Data analysis was performed using the Stat View SE Graphics program (Abacus Concepts Inc., Berkeley, CA, USA) and IBM SPSS Statistics 23 for Windows (SPSS, Chicago, IL). A *p* value <0.05 was considered as statistically significant.

## Results

Twelve patient were enrolled (Table 3). Five patients qualifying for anti-IgE treatment had received omalizumab for 6 months before recruitment, without positive clinical outcomes. After 12 months from the last BT all patients completed the clinical monitoring, but only 7 subjects accepted to repeat biopsy. No significant complications during the procedure, such as prolonged desaturations or bleeding, were registered. In one case we observed a very early complication after BT, i.e. recurrent atelectasis from fibrin plugs [14].

**Table 3:** Demographic and clinical characteristics of the asthmatic subjects included in the study

Age, years (mean ± SD/range)	54 ± 11.3/36–70
Sex (male/female	6/6
Smokers/former smokers	0/0
Atopy - number (%) of patients	5 (41.6)
FEV_1_/FVC	64.0 ± 10.7
Post-bronchodilator FEV_1_ - % predicted (mean ± SD)	77.4 ± 14.7
Inhaled corticosteroids (as mcg of beclomethasone or equivalent – range)	1000–2000
Inhaled LABA (as mcg of salmeterol or equivalent – range)	200–400
Chronic oral corticosteroid use (no - % of patients)	7–58%
Daily dose of oral corticosteroids (mg of prednisone or equivalent)	21.2 ± 13.8
Intermittent oral corticosteroids (< 6 months/year) (no - % of patients)	5–42%
Previous treatment with omalizumab - number (%) of patients	5 (41.6%)
AQLQ score at baseline (mean ± SD), 0–4 point scale	2.9 ± 0.9
ACQ score at baseline (mean ± SD), 0–7 point scale	4.7 ± 0,8
Exacerbation/patient/year (12 months before recruitment)	4.7 ± 2.0
Hospitalizations/patient/year (12 months before recruitment)	1.2 ± 1.7
Days lost of work/patient/year (12 months before recruitment)	27.2 ± 21.3

### Clinical data

**Fig. 1:**
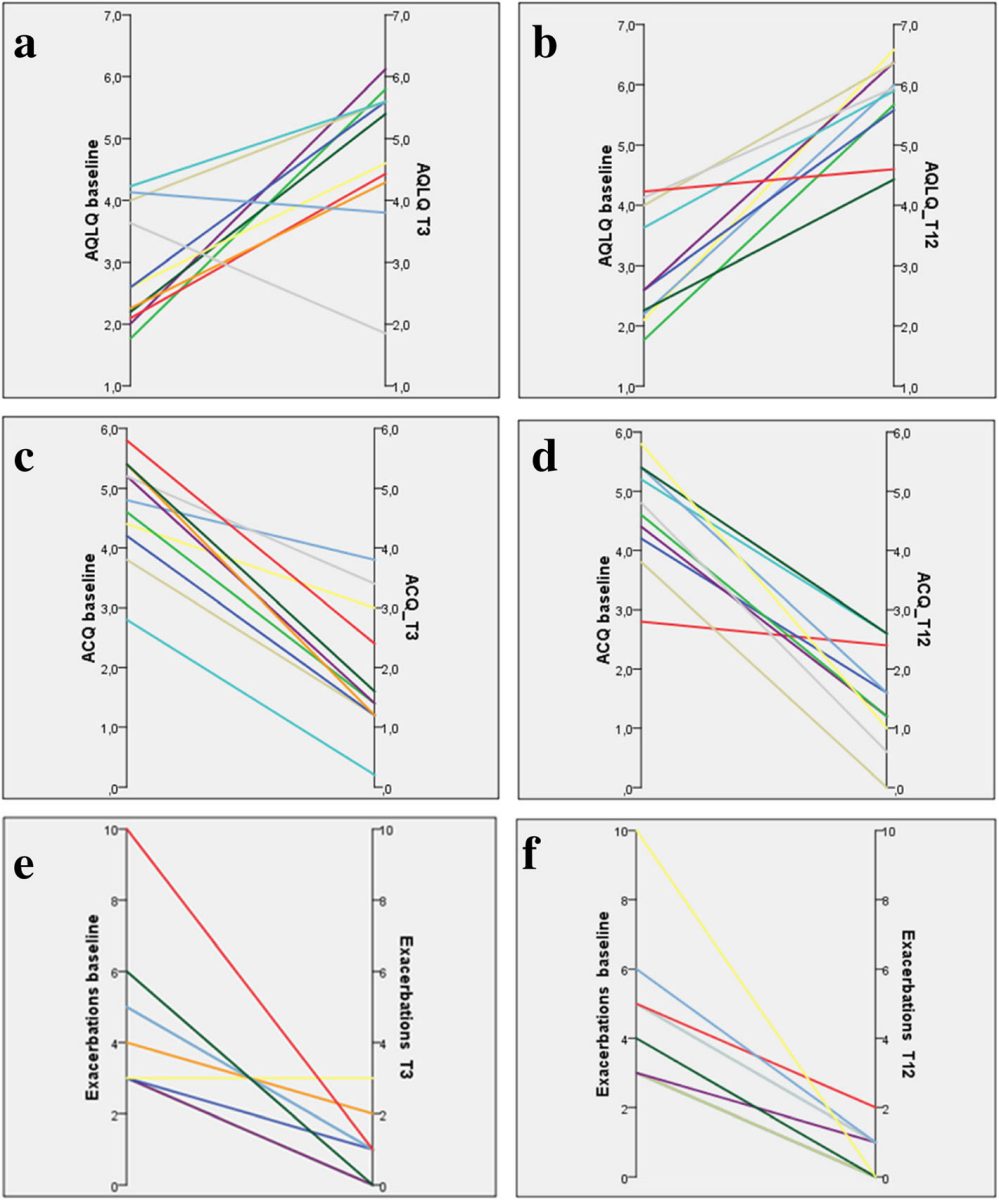
Trend of individual data (AQLQ, panels **a** and **b**, ACQ, panels **c** and **d**, exacerbations, panel **e** and **f**) at different time points: T0 vs T3, panels **a**, **c**, and **e**, T0 vs T12, panels **b**, **d** and **f**. Note that seven lines are representative of 12 patients due to overlap of data

Table 4 shows the changes in clinical parameters. A significant progressive improvement in AQLQ and ACQ was found from baseline to T3 and T12 (Fig. 1). At T3 and T12 the number of severe exacerbations was significantly reduced compared with T0. The reduction in OCS use was statistically significant at T12 (Table 4) decreasing from 21.2 ± 13.8 mg/day of prednisone or equivalent to 6.6 ± 8.6 mg/day (*p* = 0.026), with 6 patients stopping OCS. On the contrary, the bronchodilator and inhaled corticosteroid (ICS) use did not show significant changes. Exacerbations and hospitalizations dropped significantly, as shown in Table 4 and Fig. 1. The number of working days lost decreased sharply from 27.2 ± 21.3 in the year prior to BT to 0.3 ± 0.8 (*p* = 0.024, paired data test) in the year after BT. Lung function did not significantly change after BT.

**Table 4:** Clinical Characteristics of patients

	T0 (*N* = 12)	T3 (*N* = 11)	T12 (*N* = 10)	*p* value §	*p* value (T0 vs T3)	*p* value (T0 vs T12)	*p* value (T3 vs T12)
OCS – mg/ (range)	21.2 ± 13.8 (0–50)	18.7 ± 10.8 (0–25)	6.6 ± 8.6 (0–25)	0.004		0.026	0.007
FEV1% (range)	77.4 ± 14.7 (56–100)	69.0 ± 15.2 (37–97)	76.2 ± 19.6 (53–98)	0.017			
FEV1 (L) (range)	2.3 ± 0.75 (1.0–3.7)	2.0 ± 0.71 (1.0–3.3)	2.3 ± 1.11 (0.7–4.7)	0.348			
FEV1/FVC (%) (range)	64.0 ± 10.7 (47–79)	60.5 ± 13.0 (33–79)	62.8 ± 12.3 (39–79)	0.627			
RV (L) (range)	2.3 ± 1.1 (1.0–4.2)	2.1 ± 0.73 (1–4)	2.0 ± 0.66 (1.1–3.4)	0.416			
TLC (L) (range)	5.9 ± 1.5 (3.9–9.1)	5.6 ± 1.4 (3.8–8.7)	5.3 ± 1.3 (4.1–8.4)	0.322			
AQLQ (range)	2.9 ± 0.9 (1.8–4.2)	4.8 ± 1.2 (1.9–6.1)	5.7 ± 0.7 (4.4–6.6)	0.000	0.031	0.000	
ACQ (range)	4.7 ± 0.8 (2.8–5.8)	1.8 ± 1.1 (0.2–3.8)	1.5 ± 0.9 (0–2.6)	0.000	0.000	0.000	
Exacerbations/patient/year (range)	4.7 ± 2.0 (3–10)	0.9 ± 0.9 (0–3)	0.6 ± 0.7 (0–2)	0.000	0.003	0.001	
Hospitalizations/patient/year (range)	1.2 ± 1.7 (0–6)	0	0.1 ± 0.3 (0–1)	0.049			

Legend: Values are means ± SD. ^⫮^ missing data in 3 patients. *P* values are based on **§** repeated measure ANOVA (comparison among time points) and post hoc tests with Bonferroni correction (comparison between time points). Only significant values (*p* < 0.05) are reported

### Semiquantitative score of nerve fibers

**Fig. 2:**
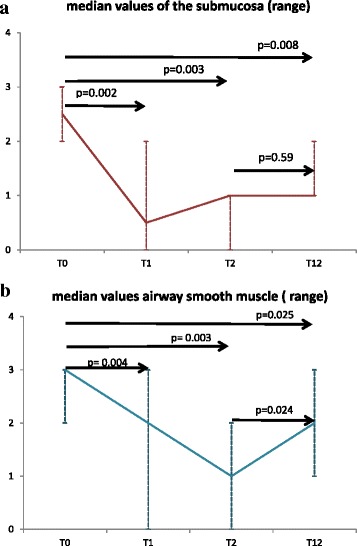
**a**-**b** X axis: time points. Y axis: nerve fiber score, 0–3 scale a) Trend of nerve fibers–PGP9.5 in submucosa (panel **a**): median and range values, T0 vs. T1 *p* = 0.002, T0 vs. T2 *p* = 0.003, T0 vs T12 *p* = 0.008 and T2 vs T12 *p* = 0.59 **b**) Trend of nerve fibers–PGP9.5 in the smooth muscle (panel **b**): median and range values, T0 vs. T1 *p* = 0.004, T0 vs. T2 p = 0.003, T0 vs T12 *p* = 0.025, and T2 vs T12 *p* = 0.024

**Fig. 3:**
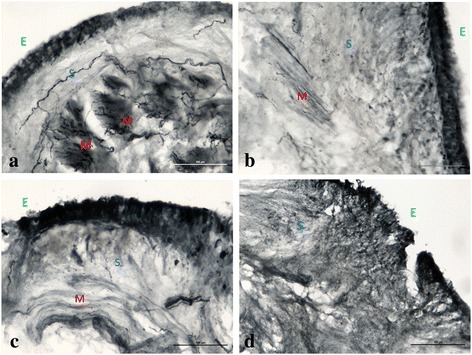
**a**) Overall picture of the nerve endings/fibers (arrows) immunodetected with PGP9.5 at time T0 in bronchial biopsies of a patient with asthma. **e**: Bronchial Epithelium (green); S: Bronchial Submucosa (blue); M: Bronchial Muscle (red). **b**) Bronchial biopsy at T1. **c**) Bronchial biopsy at T2. **d**) Bronchial biopsy at T12. Note the scarcity of nerve fibers in the submucosa and muscle bundles at T1 to T12

There was total agreement between the two independent observers in the attribution of scores. A significant reduction of total nerve fiber scores was observed in the submucosa (Friedman test *p* = 0.0003) and airways smooth muscle (*p* = 0.0003) after BT, (see Fig. 2, a and b for comparison between time points). Nerve fibers were rarely found in the epithelium, thus not allowing to perform any comparison. Bronchial glands could not be evaluated, due to technical reasons related to biopsy size (see Fig. 3).

The limited number of subjects did not allow us to evaluate possible correlations between clinical improvements (quantitative data) and variations of nerve fibers (semi-quantitative data).

Interestingly, an apparent trend to nerve regeneration in airways smooth muscle was noted given a significant increase of fibers at T12 versus T2, *p* = 0.024.

### Immunophenotype analysis of inflammatory cells

Data are reported in Table 5. The most consistent variation was found for macrophages (CD68+ cells) that were significantly higher at T1 (*p* = 0.0409), T2 (*p* = 0.0409) and T12 (*p* = 0.018) compared to T0. The number of CD4+ cells was significantly higher only at T1 (*p* = 0.0096) compared to T0. Mast-cell infiltration was significantly reduced at T1 compared to T0 (*p* = 0.0186), but not at T2 and T12 (*p* = 0.051). The degree of epithelial sloughing, the thickness of the sub-epithelial basement membrane, and the number of endothelial (CD31+) cells were similar at all time points.

**Table 5:** Structural modifications (basement membrane thickness, disepithelization degree, endothelium) and mucosal inflammatory cells in bronchial biopsies at baseline and after BT

	Friedman tes *P* value	T0	T0 vs T1 P value	T1	T0 vs T2 P value	T2	T12	T0 vs T12 P value
*Submucosa (cells/mm* ^*2*^ *)*								
*CD45*	*0.99*	*369 (253–692)*		*394 (237–796)*		*425 (313–927)*	*398 (329–492)*	
*CD4*	*0.18*	*259 (168–312)*	*0.0096*	*329 (165–548)*		*304 (167–600)*	*196 (167–304)*	
*CD8*	*0.52*	*118 (32–280)*		*130 (32–281)*		*134 (13–340)*	*196 (116–223)*	
*CD68*	*0.0024*	*16 (10–35)*	*0.0409*	*27 (13–55)*	*0.0409*	*32 (10–74)*	*61 (26–122)*	*0.018*
*Neutrophils*	*0.08*	*206 (119–393)*	*0.0499*	*250 (193–410)*		*224 (129–495)*	*220 (97–277)*	
*Eosinophils*	*0.17*	*39 (11–116)*		*58 (16–334)*		*79 (2–131)*	*211 (10–359)*	
*Mast Cells (Tryptase)*	*0.15*	*112 (32–267)*	*0.0186*	*56 (11–258)*		*77 (28–123)*	*58 (42–170)*	
*Endothelial cells-CD31*	*0.48*	*365 (240–459)*		*368 (244–597)*		*375 (341–525)*	*365 (309–455)*	
*Basement membrane thickness, μm*	*0.87*	*5.20 (4.1–7.4)*		*5.02 (3.7–7.1)*		*5.60 (3.8–7.5)*	*6.53 (5.34–8.51)*	
*% Disepithelization*	*0.404*	*72 (40–90)*		*50 (10–95)*		*70 (35–100)*	*62 (20–90)*	

Legend: Data expressed as median (range). Statistics: Friedman test for comparison among time points, Wilcoxon rank test for comparison between time points (T0 vs T1, T0 vs T2 and T0 vs T12). Only significant values (p < 0.05) are reported

## Discussion

Recently, Pretolani and colleagues demonstrated that structural components of airway wall responsible for bronchial narrowing are decreased by BT as early as at 3 months, and are associated with clinical improvements [10]. We confirm and extend this observation by showing that the nerve ablation persists at one year post-BT, supporting the hypothesis that mechanisms other than reduced amount of ASM mass may be involved in the beneficial effect of BT. The current study shows that BT induces an early and sustained decrease of nerve fiber density in the submucosa and in the airways smooth muscle. These changes were accompanied by clinical improvements.

Two efferent responses may occur through the activation of TRPV Ca^2+^ channels expressed by C-fiber nerve-endings: a centrally-mediated reflex that causes cough, bronchoconstriction, hypersecretion, tachycardia, hypotension, and a local axon reflex causing bronchoconstriction, hypersecretion, neurogenic inflammation, mast-cell activation and neuropeptide release [15–17]. West et al. demonstrated a rich amyelinated (PGP9.5-immunopositive) and myelinated (PGP9.5-immunonegative) innervation in all layers of bronchial mucosa in patients with chronic cough [18]. Recently, Pretolani et al. showed a reduction of ASM, bronchial nerve endings and neuroendocrine cells on bronchial biopsies obtained 3 months after BT in association with clinical improvement at 3 and 12 months [10]. Our study is in line with the main trials [5–7] in supporting that symptom control by BT is sustained in the long term and advocates nerve ablation as a possible alternative, or additional, mechanism of severe asthma control beside the well-known effect of BT on ASM [3, 4].

The reduction of nerve fibers in the large airways probably acts by interrupting central and local nerve reflexes responsible for the above-described effects. A recent study on the efficacy of anticholinergic agents in asthma demonstrated that tiotropium inhibits TRPV1-mediated effects of neural stimuli through a mechanism unrelated to its anticholinergic action, improves symptoms and attenuates capsaicin-induced cough in challenge studies [19].

The concept of nerve ablation for obstructive lung diseases is currently being evaluated also in COPD. Slebos DJ et al. demontrated that a novel bronchoscopic therapy (defined as targeted lung denervation) based on ablation of parasympathetic pulmonary nerves surrounding the main bronchi is feasible, safe, and well tolerated, but for clinical efficacy further investigation is needed [20].

Nerve fibers can re-generate as demonstrated in studies on lung transplantation [21]. Actually at the end of the 12 month follow up we found a trend to re-generation of fibers in airways smooth muscle (but not in the submucosa) despite no variations of clinical improvement. Future studies will have to address modifications of ASM and nerve fibers in the long term in order to fully understands the mechanism of action of BT.

In the current study, symptom control significantly improved in all patients up to 1 year after BT. OCS bursts, number of exacerbations, hospitalizations and days of work lost also decreased significantly, in agreement with what already reported in literature [5, 7, 22]. Actually, the clinical benefits observed in our small group of patients seems to exceed that reported previously in several studies of thermoplasty, but the sample is too small for a reliable comparison. Anyway, one possible suggestive explanation is that we performed a mean number of applications larger than the average in literature (75 versus 50); Langton D et al. have recently demonstrated that the number of activations delivered during BT has a role in determining clinical response to treatment [23].

At the inflammatory level, CD45+ cells did not show significant changes, demonstrating that BT has a limited impact on the global population of inflammatory cells expressing common leukocyte antigen (lymphocytes, monocytes, eosinophils). On the contrary, the number of CD4+ lymphocytes significantly increased at T1 only compared to T0. Several types of CD4+ T-cells have been implicated in asthma regulation, including Th2, regulatory T, and Natural killer (NK) T cells producing interleukin (IL)-4 and IL-13. NK and T cells in particular are steroid-resistant and may play a role in severe asthma [24]. Further studies are required on CD4 subtypes to evaluate a potential role after BT. The prevalence of neutrophils in biopsies from corticosteroid-dependent asthma patients is well known [25, 26] and was confirmed in our study. CD8+ cells and eosinophils showed a similar trend, but changes were not statistically significant. Eosinophils were on average far lower than neutrophils and mast cells, which likely depends on corticosteroid treatment.

We found a significant decrease of mast cells at T1, which was not confirmed at the subsequent time points. An absolute decrease of mast cells could be a potential explanation for the reduced frequency of bronchospasm in the short-term. A lower release of mast-cell mediators could be responsible for a down-graded functional interaction between mast cells and nerve fibers, as demonstrated in experimental studies in animal models [27].

Of interest, macrophages (CD68+ cells) progressively and significantly increased during the follow up period. In our study, macrophages were the least represented cells, as opposed to previous data on endobronchial biopsies from severe asthmatics, in which macrophages were the most predominant cell type [22]. This may be explained by different methodologies, i.e. immunohistochemistry, which is more specific than histochemistry used in other studies. Since macrophages are considered to have a potential suppressive effect on inflammation in asthma, it could be speculated that a relatively low number of macrophages is one of the biological correlates of persistent inflammation and lack of asthma control in severe patients. Conversely, an increase after BT might contribute to a better asthma control. The U-BIOPRED bronchoscopy study on asthmatics and healthy controls concluded that severe asthma persists despite suppression of endobronchial tissue inflammation in the proximal airways with regular treatment, thus reinforcing the concept that additional mechanisms in central airways are contributing to asthma severity [28]; in this sense, following our data, nerve ablation in large airways could significantly contribute to improve asthma control while the relevance of inflammatory modifications is likely negligible.

At variance with other studies [3–10], no significant structural changes were seen in terms of epithelial disruption, fibrosis and number of endothelial cells.

The effects of BT on airways smooth muscle were not evaluated in our study for two main reasons, i.e. the many scientific publications all in agreement with a reduction effect of BT on smooth muscle [3, 4, 8–10, 29–31] and a limited adequacy of bioptic specimens [32]. Data in literature confirm a persistence of ASM ablation at 27 months [29]. Our paper is the first demonstrating a persistent ablation of nerve fibers at 12 months. Whether reduction of ASM function is due to persistence of ASM ablation or reduction of nerves stimulating ASM are both plausible explanation that need further studies. The real role of the different modifications of ASM and/or nerve fiber network with respect to clinical improvement is really difficult to discriminate; at the moment, correlation between airway changes induced by BT and clinical data have been matter of debate but no definite evidence has been found.

## Conclusions

BT confirmed an important and persistent improvement of clinical outcomes at one year in patients with severe asthma. The significant reduction of nerve fibers in the submucosa and smooth muscle layers may attenuate nerve reflexes inducing bronchospasm and provides an explanation to the beneficial effects of BT alternative or complementary to the well known reduction of airways smooth muscle.

## Abbreviations

ACQ: Asthma Control Questionnaire
ANOVA: Analysis of variance
AQLQ: Asthma Quality of Life Questionnaire
ASM: Airway smooth muscle
BT: Bronchial Thermoplasty
LABA: Long-acting beta agonists
OC: Oral Corticosteroids
PGP9.5: Pan-axonal marker protein gene product 9.5
T0: Baseline
T1: One month after T0
T12: Twelve months after T2 (i.e. 14 months after baseline)
T2: Two months after T0
T3: Three months after T2
TRPV1: Transient Receptor Potential Vanilloid 1
